# Annotations

**Published:** 1902-03-29

**Authors:** 


### ANNOTATIONS.

### The Conscientious Objector.

The efforts which have been made to repeal the conscience clause in the Vaccination Act serve to draw attention to some want of uniformity in the interpretation of the clause in question. Under the section a person is not entitled to an exemption certificate by alleging simply that he has a conscientious objection, but he must "satisfy the magistrate" that he conscientiously believes that vaccination will be prejudicial to the health of his child—which is not quite the same thing. Now what will satisfy one magistrate may not go down with another; but a rule for guidance on this point has been laid down, according to which an applicant must show some grounds personal to his child, and cannot rely upon a general objection to vaccination. Thus he must produce evidence that in the case of his child the operation will be prejudicial to its health. It must be admitted that this rule rather confounds "conscientious" with "reasonable;" but the interpretation has been conceived from the point of view of the public interest; for, if the section were literally construed, the duty of vaccinating children might be too easily evaded. Fortunately the present epidemic of small-pox has removed any general danger of this kind, but there is still some risk, as is shown by an application made the other day to a London magistrate for an exemption certificate. In that case the applicant made a vague statement to the effect that he objected on principle to vaccination because of some hearsay story of injury resulting to someone therefrom. Objections on similar grounds have generally been overruled by magistrates, who have said that they were not "satisfied," etc. But in the present case the magistrate appeared to take the view that if he thought the applicant had a *bonâ fide* objection, whether reasonable or not, he was bound to grant him a certificate, though he expressed the fear which he felt that the applicant might be exposing his child to the risk of infection. It may be that the view of the meaning of the "conscience clause" is the correct one, however inimical to the public interest; but, on the other hand, an interpretation which leaves it to the conscience of each magistrate to decide whether the applicant shall have his certificate is also unsatisfactory; and the rule which has been generally acted upon, though, perhaps, not strictly defensible from the grammatical point of view, may be fully justified on public grounds.

### Unprotected Infants.

Notwithstanding the objection felt by English people to all forms of Government interference with personal concerns, it is well recognised that in some cases such interference is not only justifiable but necessary. In fact the general principle has been accepted that in cases in which infants, young persons, and even adult women, are left at the mercy of others, Government inspection is desirable. Hence all our factory and workshop legislation, and the elaborate inspection of workhouses and workhouse schools which is continually going on; hence also the Infant Life Protection Act, 1897. Such being the case it is difficult to understand, and still more difficult to sympathise with the protest which is being raised against the proposal so to amend this Act as to extend its provisions to cases in which one child only is taken in to be nursed or maintained apart from its parents. The unfortunate term "baby-farming" has led many good people to imagine that the evils connected with the custom of mothers putting out their children to be reared by strangers are confined to those cases where women make a trade of receiving a number of such infants—in fact run a "baby-farm;" that where one woman receives only one child these dangers do not exist; and that the careful inspection which is admitted to be necessary in other cases is not in this case required. We wish it were so. There is every reason, however, for believing that the secret and uninspected "one baby at a time" system is even more fatal to infant life than the more open and inspected "farms" where women make a regular trade of rearing children; and in view of the recent statement by Dr. Niven, medical officer of health for Manchester, that the death rate of illegitimate children is much more than double that of children born in wedlock (the exact numbers are 432 against 179 per 1,000) we cannot admit that it is right that the "single care" infant should be exempted from the inspection which the law provides for others. It is held by certain people who are interested in "rescue work" that the proposed amendment of the Act would make their work more difficult. We doubt, however, whether such a plea should be admitted for a moment. Indeed the very arguments raised in favour of non-inspection go far to show how necessary inspection is; the statement that non-inspected children can be put out at a lower fee than that which is asked for those under inspection, strongly suggesting that there is something wrong in the treatment of the uninspected child.

### "No Bottles."

Nothing could more markedly show the depths to which contract practice has dragged a noble profession than the interest roused by the "bottles" question when club practice is discussed. It seems that certain club doctors, although perfectly ready to give "attendance and medicine" according to their contracts, draw the line at bottles. Rallying to the battle cry, "No bottles," they defy the clubs, and hold that each club patient must provide himself with that necessary receptacle for his physic. The whole squabble is inexpressibly ignominious, and the pity of it is that the difficulty seems to be a real one; for to such a miserable remuneration has contract practice been cut down that the question of "bottles or no bottles," involves a serious proportion of such profits as the medical man derives from it. There seems every reason to believe that there are large numbers of the working classes to whom some "provident" or weekly system of payment is the only feasible method of providing themselves with medical aid in sickness, but, unless the remuneration for such contract work can be placed on a far better footing than at present, we had rather see the whole system abolished. If the payments under the present system are cut so fine that the question of bottles becomes one of serious importance, it is difficult to have much confidence in the "attendance and medicine" which are provided.

